# Glutaminolysis and Glycolysis Are Essential for Optimal Replication of Marek’s Disease Virus

**DOI:** 10.1128/JVI.01680-19

**Published:** 2020-01-31

**Authors:** Nitish Boodhoo, Nitin Kamble, Shayan Sharif, Shahriar Behboudi

**Affiliations:** a The Pirbright Institute, Woking, United Kingdom; b Department of Pathobiology, University of Guelph, Guelph, Ontario, Canada; c Faculty of Health and Medical Sciences, School of Veterinary Medicine, University of Surrey, Guildford, Surrey, United Kingdom; Northwestern University

**Keywords:** Marek’s disease virus, glucose, glutamine, virus replication

## Abstract

Viruses may hijack glycolysis, glutaminolysis, or fatty acid β-oxidation of host cells to provide the energy and macromolecules required for efficient viral replication. Marek’s disease virus (MDV) causes a deadly lymphoproliferative disease in chickens and modulates metabolism of host cells. Metabolic analysis of MDV-infected chicken embryonic fibroblasts (CEFs) identified elevated levels of metabolites involved in glutamine catabolism, such as glutamic acid, alanine, glycine, pyrimidine, and creatine. In addition, our results demonstrate that glutamine uptake is elevated by MDV-infected cells *in vitro*. Although glutamine, but not glucose, deprivation significantly reduced cell viability in MDV-infected cells, both glutamine and glucose were required for virus replication and spread. In the presence of minimum glutamine requirements based on optimal cell viability, virus replication was partially rescued by the addition of the tricarboxylic acid (TCA) cycle intermediate, α-ketoglutarate, suggesting that exogenous glutamine is an essential carbon source for the TCA cycle to generate energy and macromolecules required for virus replication. Surprisingly, the inhibition of carnitine palmitoyltransferase 1a (CPT1a), which is elevated in MDV-infected cells, by chemical (etomoxir) or physiological (malonyl-CoA) inhibitors, did not reduce MDV replication, indicating that MDV replication does not require fatty acid β-oxidation. Taken together, our results demonstrate that MDV infection activates anaplerotic substrate from glucose to glutamine to provide energy and macromolecules required for MDV replication, and optimal MDV replication occurs when the cells do not depend on mitochondrial β-oxidation.

**IMPORTANCE** Viruses can manipulate host cellular metabolism to provide energy and essential biosynthetic requirements for efficient replication. Marek’s disease virus (MDV), an avian alphaherpesvirus, causes a deadly lymphoma in chickens and hijacks host cell metabolism. This study provides evidence for the importance of glycolysis and glutaminolysis, but not fatty acid β-oxidation, as an essential energy source for the replication and spread of MDV. Moreover, it suggests that in MDV infection, as in many tumor cells, glutamine is used for generation of energetic and biosynthetic requirements of the MDV infection, while glucose is used biosynthetically.

## Abstract (precis)

Viruses can manipulate host cellular metabolism to provide energy and essential biosynthetic requirements for efficient replication. Marek’s disease virus (MDV), an avian alphaherpesvirus, causes a deadly lymphoma in chickens and hijacks host cell metabolism. This study provides evidence for the importance of glycolysis and glutaminolysis, but not fatty acid β-oxidation, as an essential energy source for the replication and spread of MDV. Moreover, it suggests that in MDV infection, as in many tumor cells, glutamine is used for generation of energetic and biosynthetic requirements of the MDV infection, while glucose is used biosynthetically.

## INTRODUCTION

Viruses are highly dependent upon the host cellular metabolism to support their replication. Glucose and glutamine are the alternative sources of carbon for energy homeostasis and biosynthesis. One molecule of glucose converts into two molecules of pyruvate and two molecules of ATP. Pyruvate enters the tricarboxylic acid (TCA) cycle and generates an additional 34 molecules (1). In tumor cells and in some virally infected cells, pyruvate is mainly converted into lactate in the cytosol, which blocks further ATP production and provides the use of glucose carbon for biosynthesis of macromolecules such as fatty acids. Both glucose and glutamine are utilized as essential sources of carbon for synthesis of macromolecules and production of energy (2–4). These processes, glycolysis and anaplerosis, may also occur in cells infected with human cytomegalovirus (HCMV), to accommodate energetic and biosynthetic requirements of viral infections (1).

Marek’s disease virus (MDV), an avian alphaherpesvirus, is a highly cell-associated virus which causes a deadly lymphoproliferative disease in chickens (5). Like all herpesviruses, MDV harbors metabolic enzymes involved in biosynthesis of energy carrier molecules facilitating early-phase infection (6–8). MDV infection modulates lipid metabolism in the infected chickens (9–12) and increases fatty acid synthesis and lipid droplet formation, which are important for virus replication (12). However, it is still unknown whether MDV-infected cells can utilize metabolites derived from fatty acid β-oxidation, an important mitochondrion and peroxisome aerobic process, for generation of energy. This process has been shown to be important in replication of Dengue virus (DENV) (13, 14).

Here, we investigated the involvement of mitochondrial fatty acid β-oxidation, using glucose and glutamine to tease out the essential requirements for MDV infection. Our results demonstrate that MDV infection increases glutaminolysis and glycolysis as well as fatty acid β-oxidation in chicken embryo fibroblast cells (CEFs). In contrast to β-oxidation, glutaminolysis and glycolysis were essential for virus replication and spread. Thus, we hypothesized that glucose and glutamine are used for production of macromolecules required for MDV infection. In MDV-infected cells, an elevated extracellular acidification rate (ECAR) suggested that pyruvate was converted into lactate. Increased MDV-induced glutaminolysis was indicated by elevated glutamine uptake and increased levels of metabolites involved in glutamine catabolism. Virus replication was rescued by α-ketoglutarate in glutamine starvation conditions, suggesting anaplerotic use of glutamine in the TCA cycle. In summary, our results suggest that glutamine catabolism is enhanced in the MDV-infected CEFs to maintain the TCA cycle, and this allows glucose to be utilized biosynthetically.

## RESULTS

### Increased levels of metabolites involved in glutaminolysis in MDV-infected CEFs.

**FIG 1:**
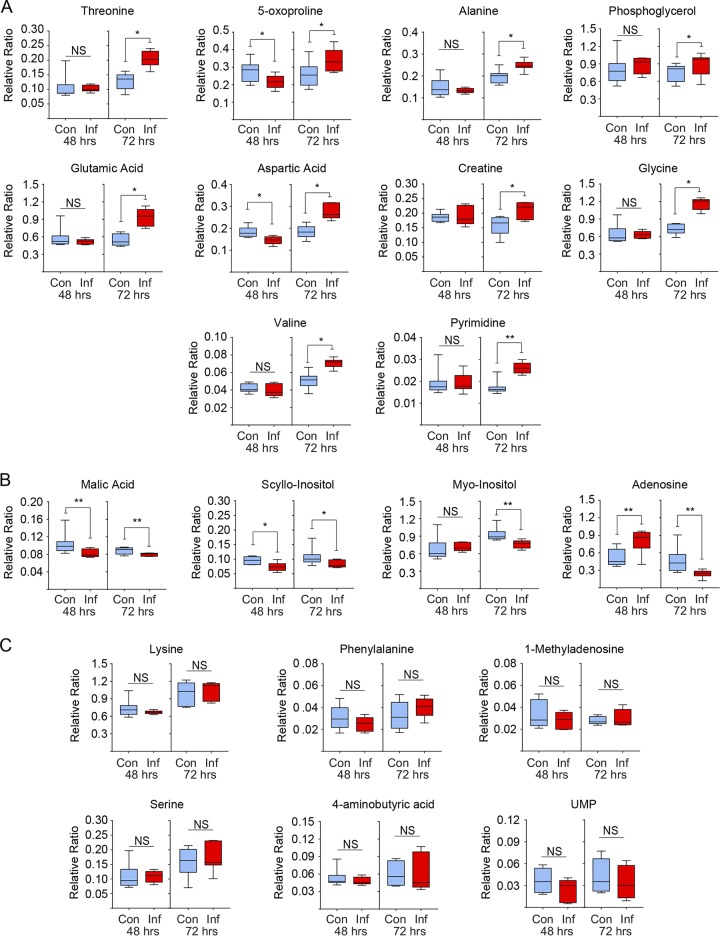
Increased levels of metabolites involved in glutaminolysis in MDV-infected cells. GSxMS analysis of relative levels of characterized metabolites from mock-infected (control) and MDV-infected (RB1B) CEFs are shown at 48 hpi and 72 hpi. Box and whisker plots showing minimum and maximum relative levels of named metabolites either (A) significantly increased, (B) significantly decreased, or (C) showed no change as a result of MDV infection. Nonparametric Wilcoxon tests (Mann-Whitney) were used to assess normal distribution and test significance, with the results shown as mean ± SD. * (*P* = 0.01) and ** (*P* = 0.001) indicate a statistically significant difference compared to the control. NS indicates no significant difference. The experiment was performed in biological triplicates with six technical replicates per biological replicates. Con, control; Inf, infected.

The relative levels of metabolites were determined in the mock- and MDV-infected CEFs at both 48 and 72 hours postinfection (hpi) using comprehensive two-dimensional gas chromatography mass spectrometry (GCxGC-MS). In total, 20 nonlipid metabolites were detected. The MDV-infected CEFs contained higher levels of 10 metabolites, including glycine and its metabolic product creatine, which are known to be involved in regeneration of cellular ATP via glutaminolysis. Higher levels of pyrimidine, glutamate (a product of glutamine deamination), alanine, and aspartic acid were also observed in the MDV-infected cells at 72 hpi (Fig. 1A). These metabolites are generally biosynthesized from glutamine during glutaminolysis. In contrast, the MDV-infected CEFs contained lower levels of four metabolites (Fig. 1B), while the levels of six other metabolites were not altered at either 48 or 72 hpi (Fig. 1C). The higher levels of glycine, creatine, alanine, and pyrimidine in the MDV-infected CEFs suggest that glutamine catabolism has been upregulated in the MDV-infected CEFs.

### Glutamine and glucose are important for maintenance of MDV infection.

**FIG 2:**
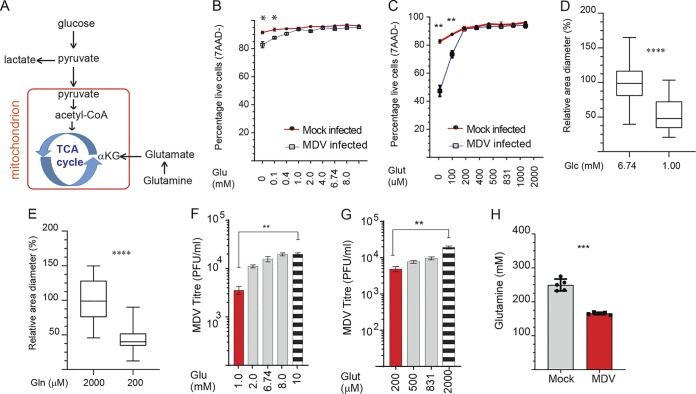
Glucose and glutamine support MDV infection. (A) Glucose and glutamine utilization in the tricarboxylic acid (TCA) cycle during MDV infection. (B and C) Cell viability of mock-infected or RB1B-infected CEFs at 72 hpi. CEFs were mock infected or infected with RB1B (100 PFU), and the cells were cultured in cell culture medium containing various concentrations of exogenous (B) glucose (0, 0.1, 0.4, 1.0, 2.0, 4.0, 6.74, 8.0, and 10 mM) or (C) glutamine (0, 100, 200, 400, 500, 831, 1,000, and 2,000 μM). Plaque sizes were determined in cell culture medium with (D) low glucose (1.0 mM) or (E) low glutamine (200 μM) or complete medium (2,000 μM and 6.74 mM glucose) at 72 hpi. Plaque sizes are shown as box plots with minimums and maximums (20 plaques were measured for each condition). Analysis of MDV viral titer (PFU/ml) in the presence of exogenous (F) glucose (1.0, 2.0, 6.74, 8.0, and 10 mM) and (G) glutamine (400, 500, 851, and 2,000 μM) at 72 hpi. (H) Glutamine levels in supernatant of mock-infected and RB1B-infected CEFs. All viral titer experiments were performed in 6 replicates, and the data are representative of 3 independent experiments. ** (*P* = 0.001) and **** (*P* < 0.0001) indicate a statistically significant difference compared to vehicle-treated cells. NS indicates no significant difference.

In glutaminolysis, glutamine is used to generate α-ketoglutarate (αKG), which can enter the TCA cycle, allowing cells to use glucose-derived carbon for biosynthetic pathways (Fig. 2A). To determine the relative importance of glucose and glutamine during MDV infection, we initially examined viability of the mock- and MDV-infected CEFs under glucose and glutamine deprivation conditions with add-back of glucose (0.1, 0.4, 1.0, 2.0, 4.0, 6.74, 8, and 10 mM) or glutamine (100, 200, 400, 500, 831, 1,000, and 2,000 μM) (Fig. 2B and C). While glucose was dispensable for optimal cell viability of noninfected CEFs, cell viability of MDV-infected CEFs was reduced by 8% in glucose-depleted medium at 72 hpi. The minimum level of glucose (1 mM) which supports high confluence and viability of MDV-infected CEFs was determined (Fig. 2B). In contrast to glucose deprivation, glutamine deprivation significantly reduced confluence and cell viability of MDV-infected CEFs, suggesting that glutamine is a major contributor of carbon that feeds the TCA cycle during MDV infection. The minimum essential level of glutamine and glucose which was required for retaining cell viability in MDV-infected cells was determined (200 μM) (Fig. 2C). MDV plaque sizes were reduced in CEFs cultured in medium containing the minimum essential levels of glucose (1 mM) or glutamine (200 μM) (Fig. 2D and E). Similarly, MDV titer was significantly reduced in the MDV-infected cells cultured in cell culture medium containing the minimum essential levels of glucose (Fig. 2F) or glutamine (Fig. 2G). Add back of glucose (1, 2.0, 6.74, 8, and 10 mM; Fig. 2F) or glutamine (200, 500, 831, and 2,000 μM; Fig. 2G) increased MDV titer in a dose-dependent manner. To demonstrate glutamine utilization in the MDV-infected CEFs, relative levels of glutamine were measured in the cell culture medium of the mock-infected and MDV-infected cells at 72 hpi. The results demonstrate a significant reduction in the levels of glutamine in the cell culture medium of MDV-infected cells (Fig. 2H), suggesting that glutamine has been utilized by MDV-infected cells. Taken together, the results indicate that both glucose and glutamine are required for MDV replication and spread.

### α-ketoglutarate, the TCA cycle intermediate, rescues MDV replication in low-glutamine conditions.

**FIG 3:**
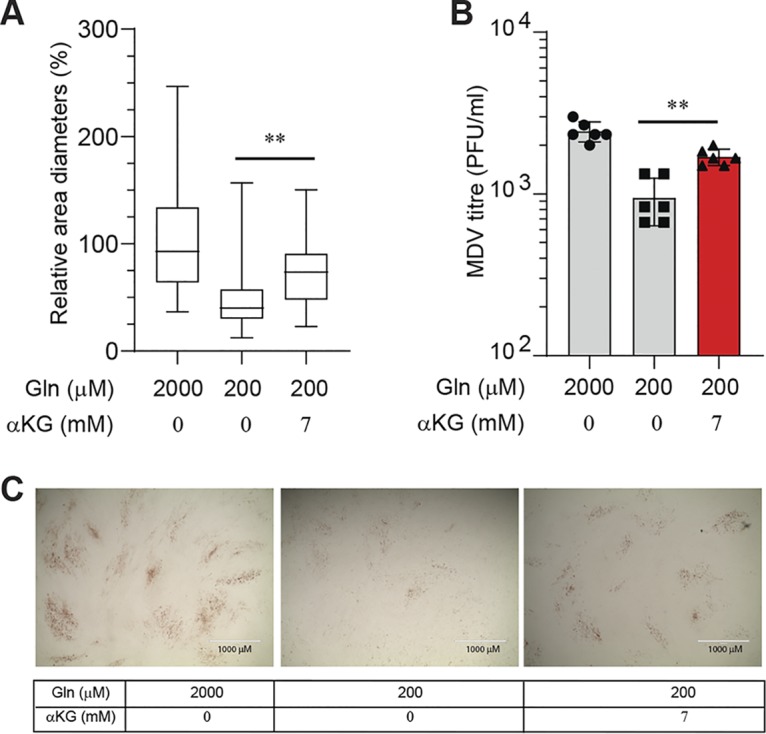
α-ketoglutarate rescues MDV replication in low-glutamine conditions. MDV-infected CEFs were cultured in complete medium (2 mM glutamine, 6.74 mM glucose) or low glutamine (200 μM) with or without α-ketoglutarate (α-KG) (7 mM). CEFs were infected with RB1B (100 PFU) and 72 hpi; (A) MDV plaque size and (B) MDV titers were determined. Plaque sizes are shown as box plots with minimums and maximums (20 plaques were measured in each well). (C) Representative plaque images are shown. Scale bar, 1,000 μm. All experiments were performed in triplicate, and data are representative of 3 independent experiments.

Glutamine can be converted to α-ketoglutarate for anaplerotic use in the TCA cycle, and this process may explain the essential role of glutamine in MDV replication. To examine the role of α-ketoglutarate in MDV replication, MDV-infected CEFs were cultured in the minimum essential level of glutamine (200 μM) in the presence or absence of nontoxic concentrations of dimethyl-α-ketoglutarate (7 mM). MDV plaque size (Fig. 3A) and virus titer (Fig. 3B) were determined at 72 hpi. The results demonstrated that α-ketoglutarate partially rescued both plaque size and virus titer. Representatives of plaque size are shown in Fig. 3C. Taken together, the results suggest that MDV-infected cells may utilize glutamine, through its conversion to α-ketoglutarate, as a TCA cycle intermediate to support MDV replication.

### MDV infection increases both oxygen consumption rate and extracellular acidification rate.

**FIG 4:**
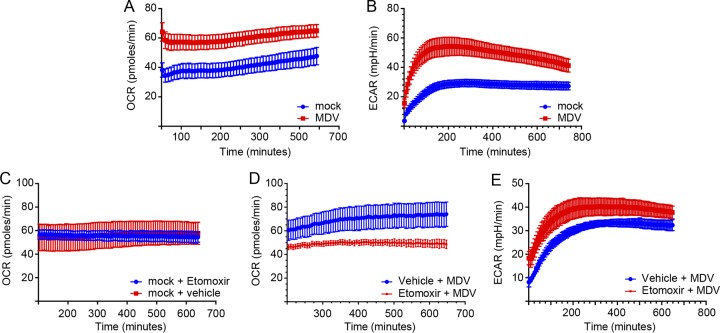
MDV infection increases OCR and ECAR. (A) Oxygen consumption rate (OCR; pmol/min) and (B) extracellular acidification rate (ECAR; mpH/min) are shown for the mock-infected and MDV-infected CEFs. (C) The OCR (pmol/min) of mock-infected CEFs treated with either etomoxir (4.42 μM) or vehicle are shown for 12 h. MDV-infected CEFs were treated with etomoxir (4.42 μM) or vehicle during the first 12 h post-mock infection, and the (D) OCR and (E) ECAR were determined using a Seahorse XFp analyzer. All experiments were performed in triplicate, and data are representative of 3 independent experiments.

The oxygen consumption rate (OCR) is increased by both glycolysis and fatty acid β-oxidation, while the extracellular acidification rate (ECAR), which was used to quantify proton production, is a surrogate marker for lactate production and glycolytic flux. Mock- or MDV-infected CEFs (72 hpi) were plated and used to determine the ECAR and OCR using a Seahorse Bioscience XFp analyzer. Linear regression analysis of the OCR and ECAR revealed a significant increase in the MDV-infected cells (Fig. 4A and B), suggesting that MDV infection increased ATP synthesis. At 600 min postrecording, the MDV-infected cells showed an OCR of 64.5 pmol/min (Fig. 4A). To assess the contribution of long-chain fatty acids to oxidative metabolism in the MDV-infected cells, we measured the OCR following the addition of a nontoxic concentration of etomoxir (4.42 μM), a small chemical inhibitor of carnitine palmitoyltransferase 1a (CPT1a) activity. Treatment of the mock-infected cells with etomoxir did not alter the OCR (Fig. 4C), while this treatment reduced the OCR in the MDV-infected cells (Fig. 4D), indicating that fatty acid β-oxidation is activated in MDV infection. In contrast, treatment of the cells with etomoxir increased the ECAR in the MDV-infected cells (Fig. 4E), suggesting that in the absence of fatty acid β-oxidation, the cells produce higher levels of lactate. Thus, the results indicate that the MDV-infected cells generate ATP via both glycolysis and β-oxidation.

### Inhibition of fatty acid β-oxidation does not reduce MDV replication.

**FIG 5:**
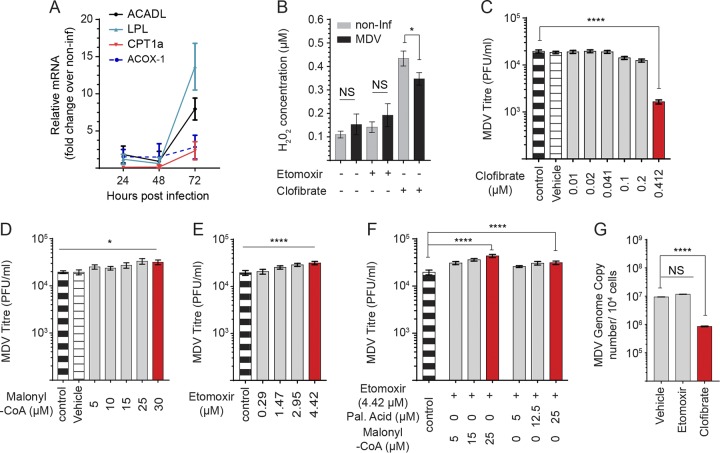
Inhibition of CPT1a increases MDV titer. (A) Fold-change expression of genes involved in fatty acid oxidation in MDV-infected CEFs at 24, 48, and 72 hpi. (B) Hydrogen peroxide (H_2_O_2_) synthesis (μM) in mock- or MDV-infected CEFs treated with etomoxir (4.42 μM) or clofibrate (0.41 μM) at 72 hpi. Analysis of MDV viral titer in MDV-infected CEFs treated with (C) clofibrate (0.01, 0.02, 0.041, 0.1, 0.2, and 0.41 μM), an agonist of PPAR-α, (D) malonyl-CoA (5, 10, 15, 25, and 30 μM), and (E) etomoxir (0.29, 1.47, 2.95, and 4.42 μM). (F) MDV viral titers are shown for MDV-infected CEFs treated with etomoxir (4.42 μM) or in combination with either palmitic acid (5, 12.5, and 25 μM) or malonyl-CoA (5, 15, and 25 μM). (G) MDV genome copy numbers per 10^4^ cells (*meq* gene with reference ovotransferrin gene) were determined using qPCR in CEFs treated with etomoxir (4.42 μM) or clofibrate (0.41 μM). Nonparametric Wilcoxon tests (Mann-Whitney) and one-way ANOVA were used to assess normal distribution and test significance with the results shown as mean ± SD. * (*P* < 0.05) and **** (*P* < 0.0001) indicate a statistically significant difference compared to the control. NS indicates no significant difference. All experiments were performed in 6 replicates for plaque assays and 3 replicates for real-time PCR and fluorometric assays. All experiments were performed in triplicate, and data are representative of 3 independent experiments.

Distinctive strategies are used by various viruses to modulate mitochondrial fatty acid catabolism and enhance viral replication (15). However, there is little information on the effects of viral infection on peroxisomal fatty acid catabolism. CPT1a is the rate-limiting step of mitochondrial fatty acid β-oxidation, and together with acyl-CoA dehydrogenase long chain (ACADL) and lipoprotein lipase (LPL) is involved in mitochondrial β-oxidation. In contrast, acyl-CoA oxidase 1 (ACOX-1), which is localized in the peroxisome, is the first enzyme involved in the synthesis of hydrogen peroxide (H_2_O_2_) (16–18). Gene expression analysis revealed upregulation of CPT1a, ACADL, LPL, and ACOX-1 mRNA transcripts in the MDV-infected CEFs at 72 hpi (Fig. 5A). In spite of β-oxidation gene transcript upregulation, MDV infection did not increase H_2_O_2_ levels (Fig. 5B), suggesting that peroxisomal fatty acid β-oxidation was not activated in MDV infection in our system. At concentrations exceeding 5 mM, etomoxir can activate reactive oxygen species and production of H_2_O_2_ (19). In this study, we used a nontoxic concentration of etomoxir (4.42 μM), which did not induce H_2_O_2_ in the mock- or MDV-infected cells. To examine the ability of peroxisomal fatty acid β-oxidation, the cells were treated with the peroxisome-proliferator activated receptor-alpha (PPAR-α) agonist clofibrate (0.412 μM), and the levels of H_2_O_2_ were measured. The results demonstrated that clofibrate significantly increased H_2_O_2_ levels in both the mock- and MDV-infected cells. Interestingly, the mock-infected cells produced higher levels of H_2_O_2_ in response to clofibrate than the MDV-infected cells (Fig. 5B), suggesting that MDV infection reduces the capability of cells to undergo peroxisomal fatty acid oxidation. Interestingly, treatment of the MDV-infected cells with clofibrate significantly reduced MDV replication (Fig. 5C), suggesting that optimal virus replication occurs when peroxisomal fatty acid β-oxidation is blocked in the infected cells. Malonyl-CoA is the physiological inhibitor of CTP1a (20), and etomoxir is the chemical inhibitor of CPT1a. To further assess the role of fatty acid β-oxidation on MDV replication, we used malonyl-CoA and etomoxir to study the role of CTP1a on MDV replication. Surprisingly, treatment of CEFs with malonyl-CoA (Fig. 5D) and etomoxir moderately increased MDV replication (Fig. 5E). A 2-fold increase was observed in MDV titers with etomoxir/malonyl-CoA or etomoxir/palmitic acid (Fig. 5F). To confirm the effects of the inhibitors on virus replication, we also quantified MDV genome copies by quantitative PCR (qPCR). As observed above, clofibrate, but not etomoxir, reduced MDV genome copy numbers (Fig. 5G). Surprisingly, our results suggest that etomoxir increases MDV titer that is independent of total MDV genome copies.

## DISCUSSION

MDV is a highly cell-associated alphaherpesvirus which infects chickens and causes a deadly lymphoma (5, 21, 22) and induces immunosuppression (23, 24). MDV infection disturbs lipid metabolism and causes atherosclerosis, which can be inhibited by vaccine-induced immunity (9, 13, 25). Chicken feather follicles are the only cells capable of producing cell-free virus; otherwise, the virus is completely cell associated (21). MDV can infect many different types of immune cells, but only CD4^+^ T cells are transformed. Lymphocytes (T and B cells) are the principal target of MDV (26), and it has been shown that lymphocytes activated by various ligands, including soluble CD40L and anti-TCRαβ antibody, can support MDV replication and transformation of CD4^+^ T cells *in vitro* (27). Most MDV-infected immune cells undergo apoptosis *in vitro* or *in vivo* (5, 28, 29), and thus, analysis of metabolic changes in lytic infected immune cells is challenging. Moreover, activation of lymphocytes by soluble CD40L and anti-TCR-αβ antibody alters cell metabolism (30), which can potentially mask the effects of MDV infection on metabolism. Unactivated human or chicken lymphocytes undergo apoptosis *in vitro*, and only cell activation by various ligands can reduce cell death in lymphocyte culture models (31, 32). Therefore, the effects of MDV infection on glycolysis and glutaminolysis were initially studied in CEFs, but these data should be confirmed in immune cells in future studies. It is important to note that CEFs, containing various cell populations including fibroblasts and immune cells, are routinely used for propagation of vaccine and pathogenic strains of MDV. Inoculum or transfection could be used to infect CEFs with MDV. In this study, we used inoculum, as transfection could potentially activate CEFs via their Toll-receptor ligands, which can lead to modulation of energy metabolism (33). The use of inoculum ensures that any metabolic alteration is solely attributed to MDV infection. In our system, over 80% of CEFs were infected at 72 hpi, which had been shown to induce maximum levels of virus titers and plaque sizes in our system. At later time points, the cells started to detach from the wells, and finally, large numbers of cell deaths were observed. These changes will affect the metabolic properties of the cells, and thus, the results from later time points will not reflect the metabolic changes exerted by the viral infection. Therefore, we decided to send samples for metabolic analysis at 48 hours and 72 hours postinfection. Based on the results, we believe that these time points truly reflect the metabolic changes in response to viral infection in CEF cells. CEF cells are routinely used for studying MDV infection *in vitro* in many laboratories, and they are the only primary cells which can be infected with MDV without a requirement for *in vitro* cell activation which will alter cell metabolism. MDV cannot transform CEF cells, and thus, virus-infected CEFs could not be used for studying metabolic changes which occur during transformation. Therefore, we believe that these cells are the only primary cells which are suited for analysis of MDV-induced cell metabolism. We had previously observed that 72 hpi is optimal timing for activation of both fatty acid synthesis and the COX-2/PGE2 pathway in MDV-infected cells (12). However, it should be emphasized that the results represent the metabolic alteration occurring in the infected cell culture, and we cannot exclude the possibility of bystander effects in which soluble factors released by the infected cells exert metabolic changes in noninfected bystander cells. Unlike immune cells, the majority of CEFs can be infected with MDV, and these cells are used to investigate the MDV replication cycle *in vitro*. In this study, we analyzed the effects of MDV infection in metabolic alteration in nontransformed cells, as metabolic changes in transformed cells may mask the effects of viral infection. However, we believe that metabolic alterations in MDV-transformed cells, which might be somehow different from MDV-infected nontransformed cells, are also important and will be explored in future studies.

Reductive carboxylation of glutamine, glutaminolysis, is an alternative pathway to fatty acid β-oxidation and aerobic glycolysis for biosynthesis of ATP (2). The mitochondrial oxygen consumption rate (OCR) is elevated when the added ADP is converted into ATP (17). Our results demonstrated that the OCR was increased in the MDV-infected CEFs, and this increase was independent of hydrogen peroxide production, a marker for fatty acid β-oxidation in peroxisomes. Alternatively, the activation of glycolysis and mitochondrial fatty acid β-oxidation can increase the OCR in cells infected with MDV. The result showing that MDV replication and spread were highly dependent on exogenous glucose levels suggests that glycolysis may provide energy and macromolecules needed for virus replication. Aerobic glycolysis is determined via measurements of the extracellular acidification rate (ECAR), which is mainly from the production of lactic acid following its conversion from pyruvate (34). Here, we observed an elevated level of ECAR, a surrogate marker for aerobic glycolysis and lactate production, in the MDV-infected cells.

Mitochondrial fatty acid β-oxidation (FAO) plays a crucial role in energy homoeostasis and oxidation of fatty acids. For the import of acyl-CoAs into the mitochondria, the carnitine shuttle relies on CPT1, which also coverts acyl-CoA into acylcarnitine (16, 17). In support of the activation of fatty acid β-oxidation in the MDV-infected cells, we demonstrated that MDV infection upregulated expression of genes involved in mitochondrial fatty acid β-oxidation, including CPT1a, ACADL, and LPL. To examine whether upregulation of these genes is associated with activation of mitochondrial β-oxidation, we determined the effects of a chemical inhibitor of CPT1a, etomoxir, on OCR. The results showed a reduction of OCR within the MDV-infected cells but not the mock-infected cells, suggesting that the elevated level of OCR is, at least partially, dependent on increased mitochondrial fatty acid β-oxidation in the MDV-infected cells. Conversely, etomoxir increased ECAR levels in the MDV-infected cells, suggesting that there is a shift in glucose catabolism, generating higher levels of lactate, in the absence of fatty acid β-oxidation. Thus, our results indicate that ATP production is increased in the MDV-infected cells via glycolysis and mitochondrial fatty acid β-oxidation in order to support virus replication. We had previously shown that MDV infection increases fatty acid synthesis (12); however, the role of fatty acid β-oxidation in MDV replication was unknown. Here, we demonstrate that fatty acid β-oxidation was not required for efficient replication of MDV, and the inhibition of β-oxidation by the CPT1a inhibitor, etomoxir, moderately increased virus titer, while it had no effect on viral copy numbers. It is still unclear how etomoxir can increase virus titer without increasing viral copy numbers. It is possible, but not proven, that etomoxir treatment increases MDV protein synthesis.

In addition to fatty acids, glucose and glutamine are the main sources of carbon for the generation of bioenergetic intermediates (2, 16, 35). We identified the minimal essential levels of glucose or glutamine that were required to maintain cell viability of the MDV-infected cells. The results demonstrate that glucose is essential for MDV replication and spread. Further work is required to determine the exact mechanism involved in the activation of glycolysis in MDV infection. In tumor cells, carbon and nitrogen atoms of glutamine, a nonessential amino acid, are used for synthesis of nucleotides, lipids, and amino acids (2, 36, 37). Glutamine is also used as an exchange factor for uptake of essential amino acids (38). Here, we demonstrated that the viability of MDV-infected cells was highly dependent on glutamine levels. This suggests that glutamine is heavily used by MDV-infected cells, and MDV replication was highly dependent on glutamine as demonstrated with low virus titer and small plaque size when minimal levels of glutamine were available. The activation of glutaminolysis in the MDV-infected cells was confirmed by our results showing higher levels of metabolites, such as bioenergetics carriers, amino acids, and glutamine derivatives, that are biosynthesized as precursors for the TCA cycle. The results revealed that the TCA cycle intermediate, α-ketoglutarate (39), partially rescued MDV titer and spread in glutamine low-cell culture conditions. This suggests that MDV switches anaplerotic substrates from glucose to glutamine. Generally, cells use glucose for ATP biosynthesis via glycolysis and the TCA cycle (35). Our results suggest that in the MDV-infected cells, glucose carbon is directed away from the TCA cycle, producing lactate and perhaps other macromolecules such as fatty acids. Thus, the use of glutamine carbon is increased to support the TCA cycle, which is required for generation of other macromolecules, such as amino acids and lipids (2). The enhanced utilization of glutamine to replenish the TCA cycle is also reported in human cytomegalovirus infection (40) and adenovirus infection (41). An increase in the level of glutamine catabolism may reduce the level of this amino acid which is crucial for efficient function of immune cells (42). Therefore, it is possible that elevated levels of glutamine catabolism in MDV infection may modulate the function of the innate and adaptive immune response against MDV. It has been shown that the alteration in glutamine catabolism by tumor cells may deplete extracellular glutamine and that this can shift the balance from Th1 response to regulatory T cells with the ability to suppress antitumor immunity (43).

The effects of serum glutamine deprivation on the survival of transforming growth factor β-positive (TGF-β^+^) regulatory T cells, which are expanded in chickens infected with MDV (23), are being examined in our laboratory. High glutamine catabolism leads to low serum concentration of glutamine, which can impair the function of immune cells, leading to poor clinical outcome and increased mortality. Low glutamine availability in viral infections reduces antioxidant protection via the glutamine-glutathione (GSH) axis and exerts a major negative impact on recovery (44). It should be noted that the *in vitro* results should be confirmed in an *in vivo* model in which glycolysis and glutaminolysis are manipulated during different stages of viral pathogenesis, and MDV replication is determined in different tissues of infected chickens. Due to the complexity of *in vivo* models, including the role of glucose and glutamine in the function of immune system cells, we believe that the *in vitro* results, presented here, will provide a better understanding of how manipulation of metabolism can directly affect MDV replication. Taken together, the results demonstrate that MDV replication depends on glucose and glutamine, and efficient MDV replication occurs when the energy is not generated via fatty acid β-oxidation.

## MATERIALS AND METHODS

### Ethics Statement.

Day-old mixed-sex specific-pathogen-free (SPF) embryonated chicken eggs were purchased from Valo (Valo Biomedia GmbH.) All embryonated chicken eggs were handled in strict accordance with the European and United Kingdom Home Office guidance and regulations under project license number 30/3169. As part of this process, the work has undergone scrutiny and approval by the ethics committee at The Pirbright Institute. Ten-day-old eggs were used to generate primary chicken embryonic fibroblast cells (CEFs).

### CEF culture and virus preparations.

CEFs were generated from mixed-sex SPF Valo eggs (Valo Biomedia GmbH) incubated in a Brinsea Ova-Easy 190 incubator at 37°C until 10 days *in ovo*. CEFs were seeded at a rate of 1.5 × 10^5^ cells/ml in 24-well plates with growth medium (E199 supplemented with 10% tryptose phosphate broth [TPB], 5% fetal calf serum [FCS], 2.8% deionized water, 0.01% amphotericin B, 10 U/ml penicillin, and 10 μg/ml streptomycin) and incubated overnight (38.5°C at 5% CO_2_). The next day, 80% confluent monolayer was observed, and growth medium was removed and replaced with maintenance medium (E199 supplemented with 10% TPB, 2.5% FCS, 3.5% SQ water, 0.01% amphotericin B, 10 U/ml penicillin, and 10 μg/ml streptomycin) or deprivation medium (E199 supplemented with 10% FCS ± glucose or glutamine). CEF cells were infected with MDV (RB1B: 40 to 100 PFU), and at 72 hpi, plaque size and virus titer were determined as described below.

### Reagents.

Clofibrate and palmitic acid (Sigma-Aldrich, Dorset, UK) were all reconstituted in dimethyl sulfoxide (DMSO). Etomoxir, malonyl-CoA, glucose, dimethyl-α-ketoglutarate, and glutamine (Sigma-Aldrich, Dorset, UK) were reconstituted in E199 medium.

### Metabolomics.

CEF cells (1.5 × 10^5^) were infected with the RB1B strain of MDV (100 PFU) in triplicate, and the cells were collected at 48 and 72 h postinfection (hpi). Some cells were kept without infection and used as control or mock-infected cells. The relative levels of different metabolites were analyzed using GCxGC-MS (Target Discovery Institute, University of Oxford), and the data were analyzed as described previously (45). The profile of detectable metabolites in the MDV- and mock-infected cells were determined in triplicate with up to six technical replicates. Based on protein content, the data were adjusted and normalized. There was no change in the size of the cells between mock- and MDV-infected cells as determined by microscopy.

### Viral plaque analysis.

Pretreatment of cells with pharmacological inhibitors: Nontoxic concentrations of palmitic acid, malonyl-CoA, etomoxir, clofibrate, and α-ketoglutarate were added to the cell monolayer 2 h prior to infection with the RB1B (100 PFU per 1.5 × 10^5^ cells).

Viral titer: MDV-infected cells were titrated onto fresh CEFs, and 72 h postinfection, the cells were fixed (1:1 acetone:methanol) and then were incubated with anti-gB monoclonal antibody (MAb) (HB-3 clone). After several washes, the cells were treated with horseradish peroxidase-conjugated rabbit anti-mouse Ig. The plaques were developed using 3-amino-9-ethylcarbazole (AEC) substrate, and viral plaques were counted using light microscopy.

Virus plaque size measurement: There were approximately 70 to 100 plaques in each well, and many plagues were fused with their adjacent plaques or were located at the edge of the wells, which made measuring their sizes very difficult. Therefore, in earlier studies, we standardized our methods and measured the sizes of all the plaques which could be measured correctly and compared that with the results from 20 plaques from each well. The data indicated that the size of 20 plaques from each well represents accurate average sizes of measurable plaques. Therefore, digital images of 20 individual plaques from each well were taken using an inverted light microscope (×4 magnification), and the pictures were processed using Adobe Photoshop software. Viral plaques were measured using the ImageJ software area tool. The results are presented as the average of plaque sizes in millimeters for each condition.

Determining nontoxic concentrations of the inhibitors: To identify nontoxic concentrations of the chemicals, mock-infected and MDV-infected CEFs were cultured in the presence of the chemicals for different times posttreatment. The cell morphology and adherence/confluence were monitored under light microscopy. Moreover, CEFs were trypsinized, stained with 7-aminoactinomycin D (7-AAD) (BD Bioscience, Oxford, UK), and acquired using MACSQuant flow cytometry, and FloJo software was used for analysis of the data. Nontoxic concentrations of the chemicals were selected based on flow cytometry data and confluence.

### qPCR to amplify MDV genes.

To amplify MDV genes, DNA samples were obtained from 5 × 10^6^ cells using the DNeasy-96 kit (Qiagen, Manchester, UK). A master mix was prepared as follows: primers Meq-FP and Meq-RP (0.4 μM), *meq* probe (0.2 μM), *ovo* forward and reverse primers (0.4 μM), and *ovo* probe (0.2 μM; 5′ Yakima Yellow/3′ TAMRA; Eurogentec) and ABsolute Blue qPCR Low ROX master-mix (Thermo Fisher Scientific, Paisley, UK). To normalize DNA samples and quantify the MDV genome copy number per 10^4^ cells, a standard curve from *meq* and *ovo* genes was utilized as described previously (46). All reactions were performed in triplicate to detect *meq* and the chicken ovotransferrin (*ovo*) gene on an ABI7500 system (Applied Biosystems).

### Real-Time PCR.

Total RNA was purified from CEFs using TRIzol (Thermo Fisher Scientific, Paisley, UK), and then cDNA was synthesized using a Superscript III first-strand synthesis kit (Thermo Fisher Scientific, Paisley, UK) and oligo-dT primers according to the manufacturer’s recommended protocol. Quantitative real-time PCR using SYBR green was performed on cDNA using the LightCycler 480 II assay (Roche Diagnostics GmbH, Mannheim, Germany). Briefly, each reaction involved preincubation at 95°C for 5 min followed by 40 cycles of 95°C for 20 s and at 55°C to 64°C (annealing temperature [T_A_] as per primer) for 15 s and elongation at 72°C for 10 s. Subsequent melt curve analysis was performed by heating to 95°C for 10 s, cooling to 65°C for 1 min, and heating to 97°C. Relative to the housekeeping gene, β-actin, relative expression levels of all genes were calculated using the LightCycler 480 software (Roche Diagnostics GmbH, Mannheim, Germany) using the primers outlined in Table 1. Data represent the mean of 6 biological replicates.

**TABLE 1:** List of primers used for real-time PCR

Gene name	GenBank accession no.	Primers^*a*^		Temmp (°C)	Product size (bp)
Lipoprotein lipase (LPL)	NM_205282	Fwd	ACTGAAACTTTTTCGCCGCTG	61	128
		Rev	ATTCATCTCAGCTTCGGGATCG		
Carnitine palmitoyl transferase 1a (CPT1a)	NM_001012898	Fwd	CTAGCCCCTCTAGCTGGCTT	61	113
		Rev	ACTTCTCTCAAGGGTTCGGT		
Acyl-coA dehydrogenase, long chain (ACADL)	NM_001006511	Fwd	CTAAGCGGCTGACTGACATCG	61	221
		Rev	AATATCCGCTCCAATGCCTCC		
Acyl-coA oxidase 1 (ACOX-1)	NM_001006205	Fwd	TTAATGACCCTGACTTCCAGC	60	167
		Rev	CCGTCCACGATGAACAAAGC		
Cytoplasmic beta actin	X00182	Fwd	TGCTGTGTTCCCATCTATCG	60	150
		Rev	TTGGTGACAATACCGTGTTCA		

a Fwd, forward; rev, reverse.

### Measurement of glutamine in culture medium.

Culture supernatant from infected (MDV RB1B, 100 PFU) and noninfected CEF cells was harvested at 72 hours. The unspent glutamine in the culture supernatant was determined using a Glutamine/Glutamate-Glo assay (Promega, USA). The harvested supernatants were analyzed as either nontreated or treated with glutaminase enzyme. The differences in relative luminescence units (RLU) between the glutaminase-treated and -nontreated groups were plotted against a standard curve. The standard curve was generated from the known quantity of glutamine (micromolar quantity) treated with glutaminase enzyme.

### Quantification of H_2_O_2_ production.

H_2_O_2_ was quantified in live cells using a fluorimetric assay (Sigma-Aldrich, Dorset, UK) according to assay kit manufacturer’s recommendation. The Red peroxidase substrate mixture (50 μl) was added directly to 96-well plates (in triplicate) at 72 hpi from CEFs either mock infected or infected with RB1B in the presence/absence of etomoxir (4.42 μM) or clofibrate (0.412 μM) and a positive control (exogenous H_2_O_2_). The reaction was allowed to proceed (38.5°C, 5% CO_2_) for 20 min in the dark. The fluorescence intensity (λ_ex_ = 540/λ_em_ = 590 nm) was measured using a multidetection luminometer (Promega, Madison, WI, USA). The concentration of H_2_O_2_ was determined against a standard curve (0 to 10 μM).

### Metabolic flux analysis.

CEFs were seeded at a rate of 2.0 × 10^4^ cells per well in an XFp 8-well V-3 polyethylene terephthalate (PET) tissue culture miniplate (Seahorse Bioscience, UK) in triplicate and incubated overnight at 38.5°C. The next day, cells at 80% confluence were pretreated with etomoxir (4.42 μM) or vehicle control and then either mock infected or infected with RB1B (100 PFU per 1.5 × 10^5^ cells) for an additional 24 h. Baseline oxygen consumption rates (OCR) were measured every 6 min using the Seahorse Bioscience XFp analyzer (Seahorse Bioscience, UK) from 16 to 28 hpi, 38 to 50 hpi, and 60 to 74 hpi at 38.5°C.

### Statistical Analysis.

All data are presented as mean ± standard deviation (SD) from at least three independent experiments. Quantification was performed using Graph Pad Prism 7 for Windows. The differences between groups in each experiment were analyzed using nonparametric Wilcoxon tests (Mann-Whitney) or the Kruskal-Wallis test (one-way analysis of variance [ANOVA], nonparametric). Results were considered statistically significant at *P < *0.05 (*).
